# Germination and Heat Resistance of *Parageobacillus* and *Geobacillus* spp. Spores

**DOI:** 10.3390/foods14122061

**Published:** 2025-06-11

**Authors:** Maika Salvador, Santiago Condón, Elisa Gayán

**Affiliations:** Department of Animal Production and Food Science, Faculty of Veterinary, AgriFood Institute of Aragon (IA2), University of Zaragoza—CITA, Miguel Servet 177, 50013 Zaragoza, Spain; maikasalvador16@gmail.com (M.S.); scondon@unizar.es (S.C.)

**Keywords:** heat resistance, germination, *Geobacillus*, *Parageobacillus*

## Abstract

*Geobacillus* and *Parageobacillus* spores are major spoilage agents in thermally treated, shelf-stable foods, particularly milk products, due to their high heat resistance. This study aimed to investigate how spore purification, maturation time, and sporulation temperature influence the germination and heat resistance of *P. thermoglucosidasius*, *G. thermodenitrificans*, and *G. stearothermophilus* spores, with the goal of improving the reliability of microbial risk assessment. All three species germinate efficiently in milk, likely triggered by lactose and glucose. Ethanol-treated spores during purification germinated without heat activation, while water-washed spores required it. At least four days of maturation were needed for efficient germination, though extending maturation to seven days led to strain-dependent changes in heat resistance: it increased in *G. thermodenitrificans*, decreased in *P. thermoglucosidasius*, and remained stable in *G. stearothermophilus*. Sporulation at 55 °C consistently favored germination at the same revival temperature. *G. stearothermophilus* reached the highest heat resistance at 55 °C, whereas the other species were more resistant when sporulated at 60 °C. These findings underscore the importance of standardizing spore-preparation protocols, as key parameters such as purification, maturation time, and sporulation temperature critically affect spore properties relevant to food stability.

## 1. Introduction

The genus *Geobacillus* and the recently reclassified *Parageobacillus* consist of spore-forming bacteria characterized by an optimal growth temperature ranging between 45 °C and 65 °C [1,2]. Despite their thermophilic trait, these bacteria are ubiquitous in nature and therefore can easily enter the food chain [1,3,4]. The *Geobacillus* and *Parageobacillus* species are common contaminants in mildly acidic self-stable foods, particularly ultra-high-temperature (UHT) processed or sterilized milk and milk powders [5,6]. Among them, *G. stearothermophilus* is one of the most prevalent bacteria in dairy products [7,8,9], although other species such as *G. thermodenitrificans* and *P. thermoglucosidasius* are also frequently isolated [10,11,12,13]. This high prevalence likely originates from spores produced in biofilms on manufacturing surfaces maintained at elevated temperatures, such as heat exchangers and evaporation sections, rather than contaminated raw milk [6,14]. Spores of *Geobacillus* and *Parageobacillus* are extremely high-heat resistant and can survive commercial thermal treatments [12,15,16,17]. The germination and subsequent growth of surviving spores during storage under warm conditions, which are becoming increasingly common due to climate change [16,18], along with the proteolytic and lipolytic enzymes produced during vegetative growth under processing conditions, can lead to off-flavors and coagulation in dairy products and their final applications [5,14,19,20].

Thermal processing conditions are often optimized to eliminate sufficient levels of thermophilic spores, particularly *G. stearothermophilus*, while minimizing negative effects on product quality [21]. Additionally, *G. stearothermophilus* spores are widely used as biological indicators for sterilization validation, not only in food processing but also in the medical and pharmaceutical industries [22]. These validation processes often involve challenge tests to assess heat resistance and/or germination in end products using spores synthetically produced under laboratory conditions.

Sporulation medium composition, incubation temperature, maturation time, and purification techniques are known to significantly influence both germination and heat resistance in model mesophilic *Bacillus* spp. spores [23,24,25,26,27,28]. However, the impact of these factors on *Parageobacillus* and *Geobacillus* spp. spores, whose germination and resistance properties widely differ from mesophilic *Bacillus* spp. spores [29,30], remains largely unexplored. Addressing this knowledge gap is essential for designing effective thermal processing strategies that accurately assess the risk of spore survival without compromising food quality.

The aim of this study was to evaluate the effects of spore-purification methods, maturation time, and sporulation temperature on the germination and heat resistance of *P. thermoglucosidasius* (DSM 2542), *G. thermodenitrificans* (DSM 465), and *G. stearothermophilus* (ATCC 12980) spores, after confirming that these three strains are representative candidates for milk spoilage.

## 2. Materials and Methods

### 2.1. Obtention and Purification of Spore Suspensions

The strains *P. thermoglucosidasius* DSM 2542, *G. thermodenitrificans* DSM 465, and *G. stearothermophilus* ATCC 12980 were provided by the Bacillus Genetic Stock Center (Columbus, OH, USA). The strains were stored at −80 °C in 2TY broth (Sigma-Aldrich, St. Louis, MO, USA) supplemented with 25% glycerol (Panreac, Barcelona, Spain). For revitalization, cells were streaked on Tryptone Soya Agar (Oxoid, Basingstoke, UK) supplemented with 0.6% yeast extract (Oxoid) (TSAYE) and incubated at 55 °C for 24 h.

For sporulation, a single colony was inoculated into a 250 mL flask containing 20 mL of 2TY broth and was incubated at 55 °C overnight with shaking (130 rpm; Heidolph Promax 1010, Schwabach, Germany). Afterwards, a volume of 200 µL from the culture was inoculated into a 500 mL Erlenmeyer flask containing 50 mL of liquid TYE sporulation medium. The composition of this medium was optimized to reach the maximum sporulation yield in the three species, and it was composed of 0.4% tryptone (VWR International Chemicals, Radnor, PA, USA), 0.4% yeast extract, 1.13 mM CaCl_2_ dihydrate (VWR International), 0.033 mM MnSO_4_ monohydrate (Carlo Erba Reagents GmbH, Milan, Italy), 1 mM MgSO_4_ heptahydrate (Panreac), 0.04 mM FeSO_4_ heptahydrate (Sigma-Aldrich), and 80 mM HEPES of pH 7.0 (Sigma-Aldrich). As reference conditions, the sporulation cultures were incubated at 55 °C with a magnetic stirring (250 rpm; MIXdrive 6, 2mag, Munich, Germany) for 4 d. To study the effect of maturation time, the spores were also harvested at 1, 2, or 7 d of incubation at 55 °C, and to study the effect of incubation temperature, sporulation was also performed at 50 °C and 60 °C for 4 d. The sporulation yield was monitored over time by phase-contrast microscopy (Nikon Eclipse E400, Tokyo, Japan), calculating the proportion of bright-phase spores over total cells, and by plate counts, estimating the difference in the number of CFU/mL before and after heat treatment at 80 °C for 15 min applied in an Eppendorf thermoblock (LABNET International, Edison, NJ, USA).

The spores were harvested by centrifugation at 3345× *g* (Gyrozen 1736R, CIC Controltecnica, Madrid, Spain) for 20 min at 4 °C, and the spores from the pellets were purified using four different procedures:

- Protocol 1: four consecutive washes with distilled water [30];
- Protocol 2: one wash with distilled water followed by ethanol treatment (50%, *v*/*v*; SAEQSA, Zaragoza, Spain) for 1 h at 25 °C and four washes with distilled water [31];
- Protocol 3: four washes with distilled water followed by Tween 80 treatment (0.01%, *v*/*v*; Sigma-Aldrich) for 1 h at 25 °C and four washes with distilled water [32];
- Protocol 4: one wash with distilled water followed by three washes with 0.1% (*v*/*v*) Tween 80, ethanol treatment (50%, *v*/*v*) for 1 h at 25 °C, three washes with 0.01% Tween 80, and four washes with distilled water [33].

None of the procedures included gradient density centrifugation due to the low levels of cell debris observed after each purification procedure and the fact that it may reduce heat resistance [25].

After each method, the spore purity (99% bright-phase spores) was verified by phase-contrast microscopy and the suspensions were kept at −20 °C until usage. To assess biological variability, three different spore populations were obtained at each environmental condition.

### 2.2. Germination Assays

Germination was monitored by the reduction in optical density at 600 nm (OD_600_) resulting from the release of dipicolinic acid (DPA) and the rehydration of spores. The germination of spore samples adjusted to an OD_600_ of 0.4–0.6 was induced by a GR-saturating concentration of glucose (100 mM), lactose (100 mM), or casein hydrolysate (2%, *w*/*v*; Merck Millipore, Rahway, NJ, USA) in 25 mM HEPES buffer (pH 7.4). Where indicated, the spores were heat-treated at 100 °C for 30 min in an Eppendorf thermoblock and then incubated on ice for 15 min before exposure to nutrients [34]. OD_600_ was measured using a multiwell plate reader (CLARIOstar Plus, BMG, Ortenberg, Germany), which automatically recorded data every 3 min. Each reading was preceded by 30 s of shaking to prevent spore sedimentation. Samples were routinely germinated for 4 h at 55 °C, but also at 50 °C and 60 °C when evaluating the interaction between sporulation and germination temperatures. Germination curves were constructed using the percentage of OD_600_ fall (OD*_t_*/OD_0_ × 100, where OD_0_ and OD*_t_* represent the initial value and the value measured at further incubation times, respectively).

At the end of the spectrophotometric assays, the percentage of germinated spores was determined by phase-contrast microscopy. A total of 100 to 150 individuals per sample were examined and categorized as either dormant (phase-bright cells) or germinated spores (phase-dark and -grey cells). The lower and upper limits of quantification for germinated spores were approximately 5.0% and 97.0%, respectively.

In addition, germination and growth was assessed in UHT whole, skim, or whole lactose-free milk (DIA Supermercados, Madrid, Spain). The spores were inoculated in an Eppendorf containing 250 µL of milk to a final concentration of ca. 3 × 10^8^ CFU/mL, and the samples were incubated at 55 °C in an Eppendorf thermoblock (LABNET International, Edison, NJ, USA) with shaking (300 rpm). Germination was estimated by the evolution of the plate count after heat treatment (80 °C, 15 min) over time (Log(*N*_0_/*N_t_*), where *N*_0_ and *N_t_* represent the number of survivors in CFU/mL at time 0 and after different intervals, respectively.

For each spore preparation and germination condition, we obtained germination curves from at least three different biological replicates.

### 2.3. Thermal Treatments

Heat treatments were conducted using a thermoresistometer TR-SC [35]. This piece of apparatus consists of a 450 mL stainless steel chamber equipped with an electric heater, a cooling coil, and a stirring mechanism to ensure the uniform distribution of inoculum and temperature. Nitrogen gas at a pressure of 2.0 bars was introduced into the treatment chamber to achieve temperatures exceeding 100 °C and facilitate sample extraction over time. Once the target temperature was stabilized (±0.1 °C), spores were introduced into the treatment chamber containing McIlvaine citrate-phosphate buffer at pH 7.0 [36], achieving a final concentration of approximately 5 × 10^4^ CFU/mL. Subsequently, 0.2 mL samples were taken at different intervals of time, and viability was assessed according to the procedure described below. For each spore preparation condition and treatment temperature, we obtained three inactivation curves from different biological replicates.

### 2.4. Determination of Viability

Viability was determined by pour-plating in TSAYE; plates were incubated at 55 °C for 48 h. Longer incubation times did not affect the survival counts. The plate counts were obtained using an automatic colony counting system by using image analysis. The limit of quantification was 1.5 × 10^2^ CFU/mL for thermal inactivation assays and 3 × 10^2^ CFU/mL for growth assays.

### 2.5. Modeling of Heat Inactivation Curves

Inactivation curves were constructed by plotting the survival fraction (Log(*N_t_*/*N*_0_)) against treatment time. As most survival curves displayed a shoulder phase, the data were fitted to the Log-linear + shoulder equation proposed by Geeraerd, et al. [37] (Equation (1)), using the GInaFiT Excel tool [38] (KU Leuven, Leuven, Belgium). In this model, *N*_0_ is the initial cell concentration (CFU/mL), *N_t_* is the cell concentration (CFU/mL) at a specific time *t*, *Sl* (shoulder length, min) is the time required to reach the exponential inactivation phase, and *K_max_* (inactivation rate, min^−1^) is the slope of the exponential portion of the survival curve. The GInaFiT Version 1.6 software also provides the coefficient of determination (R^2^) and the root mean square error (RMSE) to evaluate the goodness of fit.

(1) $$Log\;N_{t}=Log\;N_{0}-\frac{K_{max}\;t}{Ln10}+Log\left(\frac{{exp}^{K_{max}\;Sl}}{1+({exp}^{K_{max}\;Sl}-1){exp}^{-K_{max}\;t}}\right)$$

For comparison, we calculated the time needed for 3 Log reductions (3D_T_) for each treatment temperature. To evaluate the impact of treatment temperature on heat resistance, thermal death time (TDT) curves were generated by plotting Log 3D_T_ values against temperature. z values, which represent the number of degrees required to decrease the 3D_T_ value by one Log unit, were calculated as the inverse of the slope of the TDT curves.

### 2.6. Statistical Analysis

Two-way ANOVA with Tukey’s and Sidak’s multiple comparisons test and unpaired parametric *t*-test were performed using GraphPad PRISM 8.4.2 (GraphPad Software Inc., San Diego, CA, USA). Statistical significance was considered when the *p* value was ≤0.05. The data presented in the figures represent averages and standard deviations derived from a minimum of three biological replicates.

## 3. Results and Discussion

### 3.1. Germination in Milk Products

**Figure 1:**
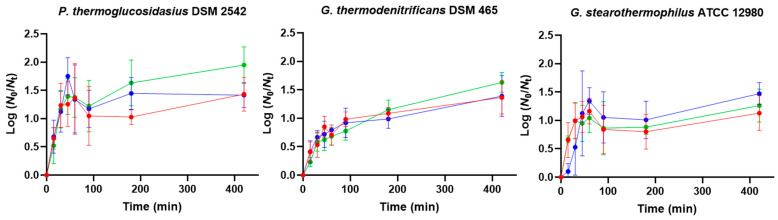
Germination kinetics of *P. thermoglucosidasius* DSM 2542, *G. thermodenitrificans* DSM 465, and *G. stearothermophilus* ATCC 12980 spores in whole milk (red dots), skim milk (blue dots), and whole lactose-free milk (green dots) at 55 °C. Spores were produced at 55 °C for 4 d and purified using ethanol treatment followed by water washes (protocol 2). Germination was determined by the decrease in plate counts after mild heat treatment (80 °C, 15 min) over time (Log(*N*_0_/*N*_t_)). Data in the figures correspond to mean values and standard deviations calculated from three biological replicates.

We investigated spore germination of *P. thermoglucosidasius*, *G. thermodenitrificans*, and *G. stearothermophilus* strains in whole milk, skim milk, and whole lactose-free milk at 55 °C to assess their potential as representative food-spoilage agents (Figure 1). *P. thermoglucosidasius* spores germinated the fastest, reaching an average of 0.62 Log CFU/mL of germinated cells (75.9% germination) after 15 min, and 1.17 Log CFU/mL (93.3%) after 30 min across all of the milk products. *G. stearothermophilus* spores germinated at a similar rate to *P. thermoglucosidasius* in whole and lactose-free milk (ca. 0.68 Log CFU/mL, 79.2%, at 15 min, and 1.05 Log CFU/mL, 91.0%, at 30 min). In skim milk, the germination of *G. stearothermophilus* spores was slower, reaching only 0.53 Log CFU/mL (70.3%) at 30 min, and increasing to 1.12 Log CFU/mL (92.5%) at 45 min. *G. thermodenitrificans* spores germinated more slowly, requiring 90 min in whole milk and 180 min in skim and lactose-free milk to exceed 90.0% germination. After 7 h, the germination efficiency was similar (*p* > 0.05) among the three strains in whole and skim milk (on average 1.37 Log CFU/mL, 95.7%). In lactose-free milk, *P. thermoglucosidasius* and *G. thermodenitrificans* reached the highest germination efficiency (1.78 Log CFU/mL, 98.5%), which was significantly (*p* ≤ 0.05) higher than that of *G. stearothermophilus* (1.26 Log CFU/mL, 94.5%). Extending incubation to 24 h did not improve germination (*p* > 0.05). It is important to note that *P. thermoglucosidasius* and, to a lesser extent, *G. stearothermophilus* proliferated in the three milk products, while *G. thermodenitrificans* could not grow (Figure S1). However, lactose-free milk contaminated with *G. thermodenitrificans* exhibited signs of spoilage (coagulation) after 24 h, as did the *P. thermoglucosidasius* and *G. stearothermophilus* samples, suggesting that some germinated spores may resume metabolic activity.

**Figure 2:**
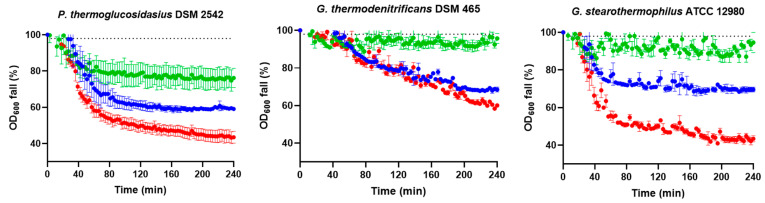
Germination kinetics of *P. thermoglucosidasius* DSM 2542, *G. thermodenitrificans* DSM 465, and *G. stearothermophilus* ATCC 12980 spores in casein hydrolysate (green dots), glucose (red dots), or lactose (blue dots) at 55 °C. Spores were produced at 55 °C for 4 d and purified using ethanol treatment followed by water washes (protocol 2). Germination was determined by the percentage of OD_600_ fall over time (OD_t_/OD_0_ × 100). Data in the figures correspond to mean values and standard deviations calculated from three biological replicates. The dotted line represents the mean OD_600_ decrease in samples with no germinants added.

To identify potential germinants, we explored germination in response to milk containing carbohydrates (lactose and glucose, the latter as a lactose breakdown product) and casein hydrolysate (an amino acid source) by measuring the OD_600_ decrease over 4 h at 55 °C. As shown in Figure 2, the three strains germinated in glucose and lactose, but not in the amino acid mixture. Only the *P. thermoglucosidasius* spores showed a significant OD_600_ decrease, but to a lower level than in both sugars. This is in contrast to the typical germination of *Bacillus* and *Clostridium* spp. spores, which germinate efficiently in the presence of certain L-amino acids [39,40]. Closer examination of the germination efficiency at the end of the 4 h assays by phase-contrast microscopy revealed that *P. thermoglucosidasius* exhibited a significantly (*p* ≤ 0.05) higher proportion of phase-dark spores than the other two species in both lactose and glucose, and that all three species germinated between 1.2- and 1.7-fold more efficiently (*p* ≤ 0.05) in glucose than in lactose.

Overall, *P. thermoglucosidasius* DSM 2542, *G. thermodenitrificans* DSM 465, and *G. stearothermophilus* ATCC 12980 spores germinated efficiently in milk products, likely triggered by lactose or, in lactose-free milk, by glucose. Interestingly, although *G. stearothermophilus* ATCC 12980 spores responded to lactose, this strain lacks the genetic capacity for lactose usage [41]. Similarly, many *P. thermoglucosidasius* isolates are unable to metabolize lactose [42,43]. However, lactose metabolism may not be essential for growth in milk. Although *G. stearothermophilus* ATCC 12980 and *G. thermodenitrificans* DSM 465 exhibited limited or no growth, respectively, in the tested dairy products (Figure S1), previous studies have demonstrated that the same strains can adhere, form biofilms, and sporulate on milk contact surfaces [10,44]. Thus, the ability of both *Geobacillus* strains to grow and sporulate in milk may depend on the physical state of the culture, in addition to variations in milk composition [45,46].

### 3.2. Effect of Purification Method on Germination and Heat Resistance

**Figure 3:**
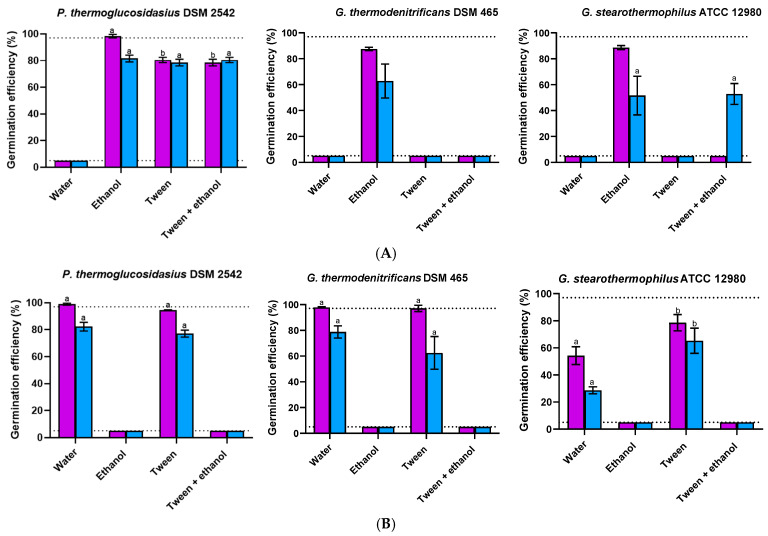
Germination efficiency of non-heat-activated (**A**) and heat-activated (**B**) spores of *P. thermoglucosidasius* DSM 2542, *G. thermodenitrificans* DSM 465, and *G. stearothermophilus* ATCC 12980 purified using different methods (protocol 1—water, protocol 2—ethanol, protocol 3—Tween, protocol 4—Tween + ethanol) after 4 h exposure to glucose (purple bars) or lactose (blue bars) at 55 °C. Sporulation was performed at 55 °C for 4 d. The dotted lines indicate the lower and upper limits of quantification (≤5.0% and ≥97.0%, respectively). Data in the figures correspond to mean values and standard deviations calculated from three biological replicates. Letters above each bar allow data to be statistically compared. There are significant differences (*p* ≤ 0.05) among spore-purification methods within each strain and germinant when they do not share the same letter.

We evaluated the effect of different spore-purification methods (for simplicity, protocol 1—water washes, protocol 2—ethanol treatment, protocol 3—Tween (0.01%) treatment, protocol 4—Tween (0.1%) washes followed by ethanol treatment and Tween (0.01%) washes) on the germination and heat inactivation kinetics of *P. thermoglucosidasius, G. thermodenitrificans,* and *G. stearothermophilus* sporulated at 55 °C for 4 d. It is important to note that none of the methods affected spore viability, as confirmed by growth on the TSAYE plate. The proportion of germinated spores after 4 h exposure to glucose or lactose, as major inducers of germination, is shown in Figure 3A. Most notably, the germination efficiency remained ≤5.0% in all populations washed only with water, but all samples subjected to ethanol treatment before water washes responded to both glucose and lactose. Additionally, the purification method had particular effects on germination in certain strains and nutrient conditions. *P. thermoglucosidasius* spores treated with Tween or subjected to Tween washes with intermediate ethanol treatment germinated in both nutrients, but the fraction of germinated spores in glucose was significantly higher (*p* ≤ 0.05) in ethanol-treated samples compared to those treated with Tween-based methods. *G. stearothermophilus* spores purified with Tween washes and intermediate ethanol treatment germinated only in lactose, achieving an efficiency comparable (*p* > 0.05) to those treated with ethanol treatment followed by water washes.

The effect of heat-activation treatment (100 °C, 30 min), previously shown to reduce the germination initiation time and increase germination efficiency in *G. stearothermophilus* NGB101 [34], was also examined (Figure 3B). Unlike non-activated spores, only heat-activated spores washed with distilled water or treated with Tween germinated in glucose and lactose, while those treated with ethanol followed by either water or Tween washes did not germinate (≤5.0%). In *P. thermoglucosidasius* and *G. thermodenitrificans*, no significant (*p* > 0.05) differences were found in germination efficiency between heat-activated spores purified with water washes or Tween treatment, regardless of the nutrient. However, in *G. stearothermophilus*, Tween-treated spores exhibited a 1.4-fold and 2.3-fold higher (*p* ≤ 0.05) proportion of germinated spores in glucose and lactose, respectively, compared to water-washed spores. Interestingly, *P. thermoglucosidasius* was the only strain in which both non-activated and heat-activated spores treated with Tween responded to nutrients. Heat activation slightly increased germination efficiency 1.2-fold (*p* ≤ 0.05; Figure 3A,B) and doubled the germinigucose but had no effect in lactose.

In conclusion, the purification method determines the response of *P. thermoglucosidasius*, *G. thermodenitrificans*, and *G. stearothermophilus* spores to nutrients and the effectiveness of prior heat activation. In general, and except for some particularities in specific nutrients and strains, spores purified with ethanol treatment followed by water washes germinated without requiring heat activation, whereas heat treatment was strictly necessary for spores prepared without ethanol exposure. Ethanol treatment (50%) is commonly used for spore purification to kill vegetative cells [31,33]. On the other hand, ethanol exposure (ca. 20–80%) has also been reported to activate germination in certain *Bacillus* and *Clostridium* species [47,48,49]. Our results suggest that ethanol purification can activate *Parageobacillus* and *Geobacillus* spores for nutrient germination, yielding efficiency comparable to heat-activated spores purified with just water washes (Figure 3).

Another intriguing finding was that ethanol-treated spores failed to germinate after heat-activation treatment. It has been suggested that sublethal ethanol and heat treatments individually enhance germination by conformational changes in essential proteins that unblock or enhance their function, with GRs thought to be the primary target in the case of heat activation, or by altering the properties of the inner membrane where such proteins reside [48,50,51]. However, ethanol treatment at an elevated temperature can damage spores, likely by disrupting spore permeability, including a reduction in inner-membrane viscosity [52,53,54]. In addition, the combined heat and ethanol treatment impairs the germination of surviving spores, presumably due to the denaturation of some critical proteins such as cortex lytic enzymes, as observed in *C. perfringens* and *B. thuringiensis* [48,55]. Thus, it can be hypothesized that transient exposure to ethanol may alter inner membrane or critical germination proteins, favoring spore germination while rendering them sensitive to subsequent heat perturbation.

The effect of the spore-purification method on heat resistance was assessed by obtaining survival curves of *P. thermoglucosidasius*, *G. thermodenitrificans*, and *G. stearothermophilus* spores prepared using the four purification protocols at a temperature appropriate for each strain (112 °C, 114 °C, and 120 °C, respectively). Table 1 displays the heat inactivation parameters, *K_max_* and *Sl*, along with the 3D_T_ values. While the purification method did not affect *G. stearothermophilus* heat resistance, *P. thermoglucosidasius* and *G. thermodenitrificans* spores exposed to Tween (with or without intermediate ethanol treatment) exhibited higher (*p* ≤ 0.05) 3D_T_ values than spores purified by the methods excluding this compound. However, these differences were minor compared to other methodological factors studied, such as maturation time and sporulation temperature (see below). Despite the fact that Tween compounds are commonly used in spore preparation to kill vegetative cells and prevent spore aggregation [32,56], to the best of our knowledge there is little information on the effect of these chemicals on spore structure. In vegetative cells, growth in media supplemented with Tween 80 or Tween 20 provides protection against acidity, high pressure, and freeze-drying [57,58,59], likely due to the incorporation of the oleic acid moiety into the cell membrane, altering its properties [57,59]. In addition, Tween compounds provide thermal stability to proteins [60,61]. Since damage to proteins related to energy metabolism—likely embedded in the inner membrane—is a key process responsible for the heat inactivation of spores, and inner membrane properties have been shown to affect heat resistance [62,63,64], it is plausible that Tween exposure may change inner membrane characteristics, thereby increasing thermal stability and thereby enhancing spore survival.

From a practical standpoint, the purification procedure of *Parageobacillus* and *Geobacillus* spores influences resistance and especially germination. The substantial impact on germination may help explain discrepancies in the literature regarding the ability of spores from the same strain to germinate in specific nutrients and the need for thermal activation [30,34,65]. However, other methodological factors, such as sporulation medium composition, the germination assay method, and the intensity of thermal treatment, may also contribute. For subsequent research, the spores were routinely purified using ethanol treatment followed by water washes, as this method rendered the spore activated for germination, avoiding the need for heat activation and without affecting heat resistance.

### 3.3. Effect of Maturation Time on Germination and Heat Resistance

**Figure 4:**
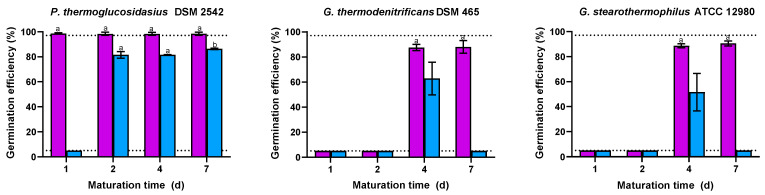
Germination efficiency of non-heat-activated spores of *P. thermoglucosidasius* DSM 2542, *G. thermodenitrificans* DSM 465, and *G. stearothermophilus* ATCC 12980 with different maturation times (1, 2, 4, or 7 d) after 4 h exposure to glucose (purple bars) or lactose (blue bars) at 55 °C. Sporulation was performed at 55 °C and spores were purified using ethanol treatment followed by water washes (protocol 2). The dotted lines indicate the lower and upper limits of quantification (≤5.0% and ≥97.0%, respectively). Values in the figures correspond to mean values and standard deviations calculated from three biological replicates. Letters above each bar allow data to be statistically compared. There are significant differences (*p* ≤ 0.05) among spores with different maturation time within each strain and germinant when they do not share the same letter.

To investigate the effect of maturation time on germination and heat resistance, spores cultured at 55 °C were collected after 1, 2, 4, or 7 d of incubation. Please note that the maximum spore yield and spore release from sporangia occurred at 24 h, with the total spore counts and viability remaining unchanged over time. Reain (Figure 4), the *P. thermoglucosidasius* spores harvested on d 1 did not germinate (≤5.0%) after 4 h exposure to lactose, while those collected between d 2 and 7 germinated in glucose and lactose to reach a similar (*p* > 0.05) efficiency. More striking was the effect of maturation time on the germination of the *Geobacillus* spp. spores. None of the spores collected on d 1 and 2 responded to glucose, whereas spores incubated for 4 to 7 d germinated to an equal (*p* > 0.05) extent. Moreover, only *G. thermodenitrificans* and *G. stearothermophilus* spores collected on d 4 were able to germinate in lactose. The requirement of a maturation period after spore release in the spent sporulation medium to detect population-wide germination, with the necessary duration depending on the strain and type of germinant, appears to be a unique characteristic of *Parageobacillus* and *Geobacillus* spores. In *B. subtilis*, the effect of maturation time has been observed at the single-cell level [26,66], but this effect may vary depending on the composition and physical state of the sporulation medium, spore age, and incubation temperature [26,67].

**Figure 5:**
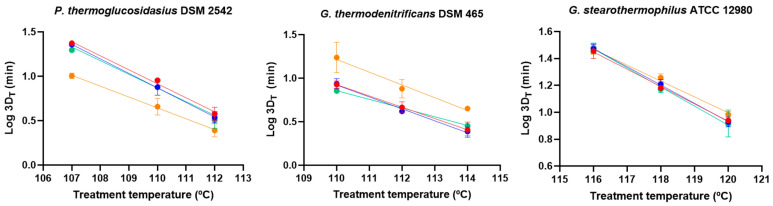
Thermal death time (TDT) curves, plotting 3D_T_ values against treatment temperature, for *P. thermoglucosidasius* DSM 2542, *G. thermodenitrificans* DSM 465, and *G. stearothermophilus* ATCC 12980 spores with different maturation times: 1 d (red dots), 2d (blue dots), 4 d (green dots), and 7 d (orange dots). Sporulation was performed at 55 °C and spores were purified using ethanol treatment followed by water washes (protocol 2). Values in the figures correspond to averages and standard deviations calculated from three biological replicates.

Maturation time also influenced heat resistance at the reference treatment temperature set for each strain (112 °C, 114 °C, and 120 °C for *P. thermoglucosidasius, G. thermodenitrificans*, and *G. stearothermophilus*, respectively). To examine the thermal dependence of this effect, inactivation kinetics were evaluated at additional temperatures (Table S1), from which TDT curves were constructed using 3D_T_ values (Figure 5). *P. thermoglucosidasius* spores incubated for 7 d exhibited lower (*p* ≤ 0.05) 3D_T_ values than those collected on d 1 and 2, with greater differences observed at lower treatment temperatures: 7 d mature spores were ca. 1.5-fold, 2.0-fold, and 2.3-fold more sensitive (*p* ≤ 0.05) than the youngest spores when treated at 112 °C, 110 °C, and 107 °C, respectively (Figure 5, Table S1). Additionally, 4 d mature spores showed lower (*p* ≤ 0.05) 3D_110 °C_ and 3D_107 °C_ values than 1 d mature spores but significantly higher heat resistance than 7 d mature spores. Consequently, z values estimated from the TDT curves increased with harvest time but not significantly (*p* > 0.05, Table 2). In contrast, *G. thermodenitrificans* spores collected on d 7 exhibited higher (*p* ≤ 0.05) 3D_T_ values than the younger spores at all of the tested temperatures (114 °C, 112 °C, and 110 °C; Figure 5, Table S1). The heat resistance of *G. stearothermophilus* spores remained unchanged with maturation time at all temperatures, except for a minor increase (1.2-fold, *p* ≤ 0.05) in 3D_118 °C_ values in 7 d mature spores compared to 1 and 4 d mature spores (Figure 5, Table S1). z values for both *G. thermodenitrificans* and *G. stearothermophilus* did not correlate with changes in maturation time (Table 2).

The observed increase in heat resistance with maturation time in the spent medium for *G. thermodenitrificans* aligns with findings for mesophilic *Bacillus* spp. spores [66,67,68,69,70]. This effect, together with changes in germination behavior, has been linked to increased cross-linking between coat proteins [66,69,71], with this structure playing a crucial role in both spore properties [72,73]. However, it remains unclear whether these changes in spore behavior are directly due to alterations in the coat structure itself, or rather to indirect effects on other components, such as the inner membrane properties or the state of embedded germination- and heat resistance-related proteins [26,74]. Additionally, Camilleri et al. [70] reported that core wet density increased with spore age, suggesting that reduced water content may also contribute to the development of heat resistance in mature spores. Further research is necessary to elucidate the relationship between changes in heat resistance with spore age and modifications in the coat, inner membrane, water content, and other structural components in each species.

Since *P. thermoglucosidasius* and both *Geobacillus* strains required at least 2 and 4 d of incubation, respectively, to germinate in lactose, and given that extending the harvest time from 4 to 7 d affected heat resistance—either increasing or decreasing it, particularly in *P. thermoglucosidasius* and *G. thermodenitrificans* spores—the maturation time for all strains was standardized to 4 d for subsequent investigations.

### 3.4. Effect of Sporulation Temperature on Germination and Heat Resistance

**Figure 6:**
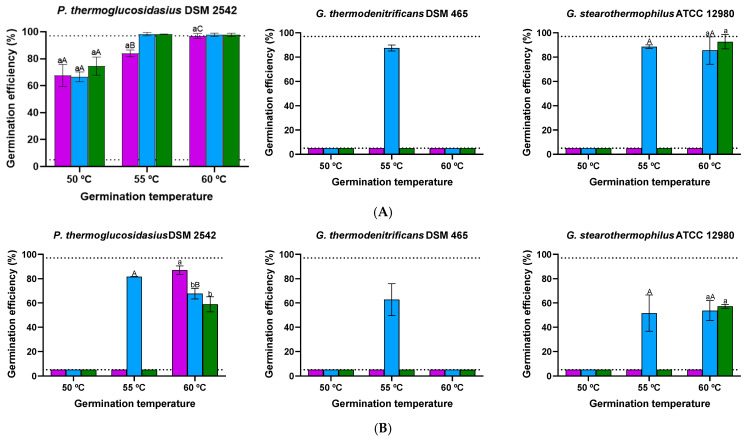
Germination efficiency of non-heat-activated spores of *P. thermoglucosidasius* DSM 2542, *G. thermodenitrificans* DSM 465, and *G. stearothermophilus* ATCC 12980 produced at different temperatures (50 °C, purple bars; 55 °C, blue bars; or 60 °C, green bars) after 4 h exposure to glucose (**A**) or lactose (**B**) at germination temperatures of 50 °C, 55 °C, or 60 °C. Sporulation was performed for 4 d, and spores were purified using ethanol treatment followed by water washes (protocol 2). The dotted lines indicate the lower and upper limits of quantification (≤5.0% and ≥97.0%). Values in the figures correspond to averages and standard deviations calculated from three biological replicates. Letters above each bar allow data to be statistically compared. There are significant differences (*p* ≤ 0.05) among spores produced at different temperatures at each germination temperature, strain, and germinant when they do not share the same lowercase letter, while there are significant differences (*p* ≤ 0.05) among germination temperatures within spores produced at each sporulation temperature, strain, and germinant when they do not share the same capital letter.

To examine the impact of sporulation temperature, spores were produced at 50 °C, 55 °C, and 60 °C. The sporulation rate and spore release were minimally affected by temperature changes, ensuring consistent maturation times. Although sporulation temperature did not affect spore viability, it did influence germination efficiency of the first germinating individuals in glucose and lactose when revived at 55 °C (Figure 6). Only spores produced at 55 °C exhibited a response (>5.0%) after 4 h exposure to glucose or lactose, except for *P. thermoglucosidasius* spores prepared at 50 °C and 60 °C in glucose. In this scenario, the germination efficiency of *P. thermoglucosidasius* spores produced at 55 °C and 60 °C exceeded 97.0%, while spores cultured at 50 °C reached a lower extent (84.0%).

As the effect of sporulation temperature may depend on the germination temperature [75,76], the response to glucose and lactose was also tested at 50 °C and 60 °C (Figure 6). Lowering the germination temperature from 55 °C to 50 °C significantly (*p* ≤ 0.05) decreased the efficiency of all *P. thermoglucosidasius* spores in glucose, while the differential effect of sporulation temperature vanished. It also inhibited (≤ 5.0%) the germination of *P. thermoglucosidasius* spores in lactose and *Geobacillus* spp. spores in both nutrients. Increasing the germination temperature from 55 °C to 60 °C enhanced the germination of *P. thermoglucosidasius* spores produced at 50 °C in glucose. It also stimulated the response of *P. thermoglucosidasius* spores prepared at 50 °C and 60 °C in lactose, with the fraction of germinated spores produced at 50 °C being approximately 1.4-fold higher (*p* ≤ 0.05) than in the other two populations. No germination (≤5.0%) was detected at 60 °C in *G. thermodenitrificans* spores, including those produced at 55 °C. Overall, sporulation temperature is a key factor influencing germination, with effects that vary depending on the strain, germinant type, and germination temperature. Generally, the three species exhibited the highest germination efficiency when sporulated and germinated at a moderate thermophilic temperature (55 °C). In *B. subtilis*, changes in germination kinetics induced by variations in sporulation temperature have been attributed to differences in active GR or GerD levels [27], alterations in the coat structure [72,77,78] and/or variations in the inner membrane properties [79,80]. For *P. thermoglucosidasius* and *G. stearothermophilus*, the germination of spores produced at temperatures lower or higher than 55 °C was enhanced by increasing the germination temperature in the presence of either lactose or glucose, but not in *G. thermodenitrificans*. The increase in germination efficiency with incubation temperature observed in *P. thermoglucosidasius* and *G. stearothermophilus* spores has also been reported for other *Bacillus* spp., where it has been hypothesized that elevated temperatures enhance molecular motion and membrane permeability, thus facilitating germination [81]. More research is needed to fully elucidate the effects of sporulation temperature on germination in mesophilic *Bacillus* spp. and, by extension, *Parageobacillus* and *Geobacillus* spp. spores.

**Figure 7:**
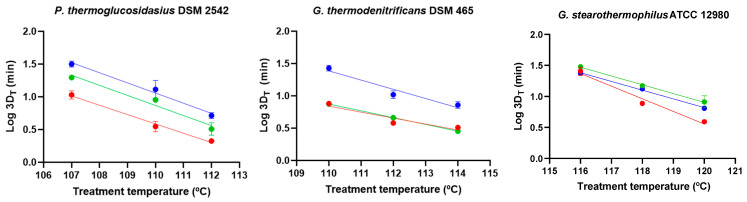
Thermal death time (TDT) curves, plotting 3D_T_ values against treatment temperature, for *P. thermoglucosidasius* DSM 2542, *G. thermodenitrificans* DSM 465, and *G. stearothermophilus* ATCC 12980 spores prouced at different temperatures: 50 °C (red dots), 55 °C (green dots), and 60 °C (blue dots). Sporulation was performed for 4 d, and spores were purified using ethanol treatment followed by water washes (protocol 2). Values in the figures correspond to averages and standard deviations calculated from three biological replicates.

As the sporulation temperature was expected to significantly influence heat resistance, inactivation curves were obtained at different treatment temperatures, and the TDT curve was generated from 3D_T_ values for each strain (Figure 7, Table S2). The 3D_T_ values of *P. thermoglucosidasius* spores increased progressively (*p* ≤ 0.05) with sporulation temperature across all treatment temperatures (Figure 7, Table S2), following similar (*p* > 0.05) z values (Table 2). In *G. thermodenitrificans*, spores produced at 60 °C exhibited the greatest (*p* ≤ 0.05) resistance at all treatment temperatures (Figure 7, Table S2). The z values of *G. thermodenitrificans* spores prepared at 60 °C were significantly (*p* ≤ 0.05) lower than those sporulated at 50 °C (Table 2). Conversely, *G. stearothermophilus* spores produced at 55 °C displayed the highest (*p* ≤ 0.05) heat resistance at all temperatures, and spores prepared at 60 °C showed greater (*p* ≤ 0.05) resistance at 120 °C and 118 °C than those sporulated at 50 °C (Figure 7, Table S1). In agreement z values of spores produced at 50 °C were lower than those sporulated at 55 °C and 60 °C.

In general, heat resistance in spore-forming bacteria, including thermophilic spores, increases with rising sporulation temperature up to a certain threshold, beyond which it either plateaus or declines [82,83,84]. Indeed, it is well established that the specific temperature range in which heat resistance positively correlates with sporulation temperature varies between species and strains [29,84,85]. The findings of this study are consistent with this trend. In *G. stearothermophilus*, spores produced at 55 °C exhibited greater heat resistance than those sporulated at 50 °C or 60 °C, which aligns with the results of Mtimet et al. [82], who reported that the highest heat resistance for the same strain (ATCC 12980) occurs at sporulation temperatures near 57 °C. In contrast, spores of *P. thermoglucosidasius* and *G. thermodenitrificans* showed increased heat resistance when the sporulation temperature increased from 50 °C or 55 °C to 60 °C. Further experimental work is needed to determine whether additional increases in sporulation temperature led to a peak in heat resistance, followed by a decline in these two strains. Leguérinel et al. [29] reported that the rate of increase in heat resistance with sporulation temperature (i.e., the increase in sporulation temperature required to change D_T_ values 10-fold) in *G. stearothermophilus* ATCC 12980 spores was higher than in other strains of *B. subtilis, B. cereus*, and *B. licheniformis*. It would be worthwhile investigating whether the magnitude of the dependence of heat resistance on sporulation temperature is similar in thermophilic microorganisms such as *P. thermoglucosidasius* and *G. thermodenitrificans*.

According to our results, the inactivation temperature should be carefully considered when comparing the effects of sporulation conditions on different strains. While variations in sporulation temperature did not affect the z value of *P. thermoglucosidasius* spores, it did influence those of *G. stearothermophilus* and *G. thermodenitrificans* spores. It is important to note that other researchers did not observe a dependency of the z value of *G. stearothermophilus* spores, including the strain used in this study, on sporulation temperature [29,86]. This discrepancy may be attributed to the influence of other environmental factors on the interaction between heat resistance and sporulation temperature, such as sporulation medium and treatment medium composition [21,85,86,87,88], as well as differences in the calculation methods used for heat-resistance parameters. Regarding the latter, z values can change when varying the Log reduction target in non-linear inactivation kinetics due to the differing weight of the shoulder length and inactivation rate [38]. Moreover, the relationship between shoulder length and inactivation rate depends on the sporulation temperature [86].

Therefore, sporulation temperature modulates heat resistance, with the extent of this effect varying depending on the strain and the treatment temperature. Heat resistance increased with the sporulation temperature in *P. thermoglucosidasius* and *G. thermodenitrificans*, while in *G. stearothermophilus*, the highest resistance was observed at the moderate sporulation temperature. The increased heat resistance with sporulation temperature in mesophilic *Bacillus* spp. has been linked to reduced core water content, elevated mineral and DPA levels, and/or structural modifications in the cortex and coat [69,73,84,89,90,91]. Therefore, investigating these characteristics in thermophilic spores could provide valuable insights.

Altogether, based on previous studies, mainly on model *Bacillus* spp. spores, it can be speculated that changes in coat structure, inner membrane properties, and/or reduced core water content may underlie the increased heat resistance associated with maturation time in *G. thermodenitrificans*, as well as the enhanced resistance observed with sporulation temperatures up to 60 °C in both *G. thermodenitrificans* and *P. thermoglucosidasius*. Additionally, it can be hypothesized that shorter maturation times and lower sporulation temperatures may alter the levels of active GRs and/or other structures, such as the coat and inner membrane, making spores less responsive to nutrients, despite ethanol treatment during purification. This suggests that such activation treatment alone may be insufficient to overcome other critical alterations affecting spore germination. Further research is needed to mechanistically elucidate the spore components driving behavioral changes under each studied factor, as well as to understand the variability in these effects among different strains. In addition, it would be interesting to investigate the interactions between purification, maturation time, and sporulation temperature at both phenotypic and mechanistic levels.

## 4. Conclusions

To the best of our knowledge, this is the first study to systematically examine how sporulation conditions, specifically temperature and maturation time, as well as spore-purification methods influence the germination and heat resistance of various *Parageobacillus* and *Geobacillus* species. Although these factors have been extensively studied in *Bacillus* spp., limited information is available for thermophilic spore-formers. Thus, our findings provide new insights into the behavior of *Parageobacillus* and *Geobacillus* spp. spores, which is particularly relevant given the anticipated increase in food spoilage caused by thermophilic spores due to climate change, as well as the growing interest in exploiting these bacteria in the biotechnology industry.

This study demonstrates that the spore purification method, maturation time, and sporulation temperature influence spore germination and heat resistance. These parameters should be carefully considered when designing challenge tests for research or industrial validation purposes. Based on representative strains of *P. thermoglucosidasius*, *G. thermodenitrificans*, and *G. stearothermophilus*, we propose general recommendations for spore preparation. However, it should be noted that the impact of each factor may vary depending on additional methodological conditions, such as the composition of the sporulation medium and spore storage conditions, as well as intraspecific variations.

For germination studies, ethanol purification followed by water washes is recommended, as it promotes efficient nutrient-induced germination without requiring heat activation. A minimum maturation period of 4 d in spent sporulation medium is necessary to obtain germination-competent spores. Optimal germination was observed when spores were produced at 55 °C and germinated at the same temperature. However, the limited germination response of *P. thermoglucosidasius* and *G. stearothermophilus* spores produced at suboptimal sporulation temperatures (50 °C or 60 °C) could be partially offset by increasing the germination temperature from 55 °C to 60 °C.

For heat resistance assays, the purification method had no effect, but increasing the maturation time had strain-specific outcomes: resistance increased in *G. thermodenitrificans*, decreased in *P. thermoglucosidasius*, and remained stable in *G. stearothermophilus*. The sporulation temperature also had strain-dependent effects: *G. stearothermophilus* spores were most resistant at 55 °C, while *P. thermoglucosidasius* and *G. thermodenitrificans* showed highest resistance when sporulated at 60 °C.

## Figures and Tables

**Table 1:** Heat resistance parameters (*Sl*, *K_max_*, and 3D_T_) of *P. thermoglucosidasius* DSM 2542, *G. thermodenitrificans* DSM 465, and *G. stearothermophilus* ATCC 12980 spores purified by different methods (protocol 1—water, protocol 2—ethanol, protocol 3—Tween, protocol 4—Tween + ethanol). Sporulation was performed at 55 °C for 4 d. Data in brackets represent the standard deviations of the mean values calculated from three biological replicates.

Strain	Treatment Temperature (°C)	Purification Method	*Sl* (min)	*K_max_* (min^−1^)	3D_T_ (min)	R^2^	RMSE
*P. thermoglucosidasius* DSM 2542	112	Water	0.57 ^a^ (0.08)	2.47 ^a^ (0.58)	3.35 ^a^ (0.40)	0.990	0.125
		Ethanol	0.12 ^b^ (0.07)	2.29 ^ab^ (0.64)	3.23 ^a^ (0.75)	0.988	0.151
		Tween	0.27 ^ab^ (0.53)	1.61 ^b^ (0.21)	4.57 ^b^ (0.57)	0.970	0.414
		Tween + ethanol	1.13 ^c^ (0.23)	1.88 ^b^ (0.09)	4.81 ^b^ (0.07)	0.973	0.351
*G. thermodenitrificans* DSM 465	114	Water	1.21 ^a^ (0.10)	4.61 ^a^ (1.09)	2.72 ^a^ (0.28)	0.988	0.133
		Ethanol	1.25 ^a^ (0.16)	4.33 ^a^ (0.20)	2.85 ^a^ (0.11)	0.981	0.280
		Tween	0.51 ^a^ (0.99)	2.87 ^b^ (0.88)	3.41 ^b^ (0.38)	0.989	0.136
		Tween + ethanol	0.32 ^a^ (0.75)	1.97 ^b^ (0.43)	4.47 ^b^ (1.23)	0.982	0.148
*G. stearothermophilus* ATCC 12980	120	Water	2.32 ^a^ (0.32)	1.90 ^a^ (0.35)	6.12 ^a^ (0.82)	0.984	0.145
		Ethanol	2.15 ^a^ (0.86)	1.16 ^a^ (0.02)	8.18 ^a^ (1.77)	0.957	0.248
		Tween	2.05 ^a^ (0.36)	1.27 ^a^ (0.35)	7.57 ^a^ (1.03)	0.965	0.333
		Tween + ethanol	2.23 ^a^ (0.56)	1.22 ^a^ (0.07)	7.91 ^a^ (0.83)	0.983	0.145

^a, b, c^ Letters allow data to be statistically compared. In each resistant parameter, there are significant differences (*p* ≤ 0.05) among spore-purification methods within each strain when they do not share the same letter.

**Table 2:** z values calculated from TDT curves (Figure 5 nr *P. thermoglucosidasius* DSM 2542, *G. thermodenitrificans* DSM 465, and *G. stearothermophilus* ATCC 12980 spores produced at different maturation times (1, 2, 4, or 7 d) and temperatures (50 °C, 55 °C, or 60 °C). Sporulation was performed at 55 °C and spores were purified using ethanol treatment followed by water washes (protocol 2). Data in brackets represent the standard deviations of the mean values calculated from three biological replicates.

Strain	Maturation Time (d)	Sporulation Temperatura (°C)	z (°C)	R^2^
*P. thermoglucosidasius* DSM 2542	1	55	6.22 ^a^ (0.69)	0.962
	2	55	6.12 ^a^ (0.33)	0.979
	4	55	6.54 ^a^ (0.92)	0.964
	7	55	8.22 ^a^ (1.23)	0.990
	4	50	7.03 ^a^ (0.59)	0.974
	4	60	6.48 ^a^ (0.83)	0.958
*G. thermodenitrificans* DSM 465	1	55	7.86 ^ab^ (1.73)	0.990
	2	55	7.36 ^a^ (1.23)	0.986
	4	55	9.78 ^bc^ (0.88)	0.996
	7	55	6.98 ^a^ (1.70)	0.948
	4	50	10.83 ^c^ (0.63)	0.877
	4	60	7.03 ^a^ (0.54)	0.914
*G. stearothermophilus* ATCC 12980	1	55	8.21 ^a^ (0.41)	0.993
	2	55	7.38 ^a^ (0.67)	0.990
	4	55	7.08 ^a^ (1.10)	0.958
	7	55	8.47 ^a^ (0.49)	0.992
	4	50	5.07 ^b^ (0.49)	0.925
	4	60	7.11 ^a^ (0.21)	0.992

^a, b, c^ Letters allow data to be statistically compared. There are significant differences (*p* ≤ 0.05) among spores of different maturation times and sporulation temperatures within the same strain when they do not share the same letter.

## Data Availability

The original contributions presented in the study are included in the article/Supplementary Material, further inquiries can be directed to the corresponding author.

## Supplementary Materials

The following supporting information can be downloaded at: https://www.mdpi.com/article/10.3390/foods14122061/s1, Figure S1. Growth curves of *P. thermoglucosidasius* DSM 2542, *G. thermodenitrificans* DSM 465, and *G. stearothermophilus* ATCC 12980 spores in whole milk (●), skim milk (●), and whole lactose-free milk (●) at 55 °C. Data in the figures correspond to mean values and standard deviations calculated from three biological replicates. The dotted line represents the limit of quantification (3 × 10^2^ CFU/mL); Table S1. Heat resistance parameters (*Sl*, *K_max_*, and 3D_T_) of *P. thermoglucosidasius* DSM 2542, *G. thermodenitrificans* DSM 465, and *G. stearothermophilus* ATCC 12980 spores with different maturation times (1, 2, 4, or 7 d). Sporulation was performed at 55 °C and spores were purified using ethanol treatment followed by water washes (protocol 2). Data in brackets represent the standard deviations of the mean values calculated from three biological replicates. Different letters indicate statistically significant differences (*p* ≤ 0.05) in resistance parameters among spores of different ages within each strain and treatment temperature; Table S2. Heat resistance parameters (*Sl*, *K_max_*, and 3D_T_) of *P. thermoglucosidasius* DSM 2542, *G. thermodenitrificans* DSM 465, and *G. stearothermophilus* ATCC 12980 spores produced at different temperatures (50 °C, 55 °C, and 60 °C). Spores were collected at day 4 and purified using ethanol treatment followed by water washes (protocol 2). Data in brackets represent the standard deviations of the mean values calculated from three biological replicates. Different letters indicate statistically significant differences (*p* ≤ 0.05) in resistance parameters among spores prepared at different temperatures within each strain and treatment temperature.

## References

[1] Najar I.N., Nagendra T. (2020). A systematic review of the genera *Geobacillus* and *Parageobacillus*: Their evolution, current taxonomic status and major applications. Microbiology.

[2] Aliyu H., Lebre P., Blom J., Cowan D., De Maayer P. (2016). Phylogenomic re-assessment of the thermophilic genus *Geobacillus*. Syst. Appl. Microbiol..

[3] Zeigler D.R. (2014). The Geobacillus paradox: Why is a thermophilic bacterial genus so prevalent on a mesophilic planet?. Microbiology.

[4] Iacumin L., Pellegrini M., Colautti A., Orecchia E., Comi G. (2022). Microbial Characterization of Retail Cocoa Powders and Chocolate Bars of Five Brands Sold in Italian Supermarkets. Foods.

[5] Burgess S.A., Lindsay D., Flint S.H. (2010). Thermophilic bacilli and their importance in dairy processing. Int. J. Food Microbiol..

[6] André S., Vallaeys T., Planchon S. (2017). Spore-forming bacteria responsible for food spoilage. Res. Microbiol..

[7] Sadiq F.A., Li Y., Liu T., Flint S., Zhang G., He G. (2016). A RAPD based study revealing a previously unreported wide range of mesophilic and thermophilic spore formers associated with milk powders in China. Int. J. Food Microbiol..

[8] Yuan D.-D., Liu G.-C., Ren D.-Y., Zhang D., Zhao L., Kan C.-P., Yang Y.-Z., Ma W., Li Y., Zhang L.-B. (2012). A survey on occurrence of thermophilic bacilli in commercial milk powders in China. Food Control.

[9] Delaunay L., Cozien E., Gehannin P., Mouhali N., Mace S., Postollec F., Leguerinel I., Mathot A.-G. (2021). Occurrence and diversity of thermophilic sporeformers in French dairy powders. Int. Dairy J..

[10] Karaca B., Karakaya A.B., Ozcan B., Coleri Cihan A. (2022). Rapid detection of *Geobacillus* and *Anoxybacillus* species by quantitative qPCR (qPCR) in commercial dairy products. J. Food Saf..

[11] Karaca B., Buzrul S., Coleri Cihan A. (2019). *Anoxybacillus* and *Geobacillus* biofilms in the dairy industry: Effects of surface material, incubation temperature and milk type. Biofouling.

[12] Sadiq F.A., Flint S., He G. (2018). Microbiota of milk powders and the heat resistance and spoilage potential of aerobic spore-forming bacteria. Int. Dairy J..

[13] Manachini P.L., Mora D., Nicastro G., Parini C., Stackebrandt E., Pukall R., Fortina M.G. (2000). *Bacillus thermodenitrificans* sp. nov., nom. rev. Int. J. Syst. Evol. Microbiol..

[14] Murphy S.C., Martin N.H., Barbano D.M., Wiedmann M. (2016). Influence of raw milk quality on processed dairy products: How do raw milk quality test results relate to product quality and yield?. J. Dairy Sci..

[15] Pujol L., Albert I., Magras C., Johnson N.B., Membré J.M. (2015). Probabilistic exposure assessment model to estimate aseptic-UHT product failure rate. Int. J. Food Microbiol..

[16] Koutsoumanis K.P., Misiou O.D., Kakagianni M.N. (2022). Climate change threatens the microbiological stability of non-refrigerated foods. Food Res. Int..

[17] Peñalver-Soto J.L., Garre A., Aznar A., Fernández P.S., Egea J.A. (2021). Dynamics of Microbial Inactivation and Acrylamide Production in High-Temperature Heat Treatments. Foods.

[18] Kakagianni M., Koutsoumanis K.P. (2018). Mapping the risk of evaporated milk spoilage in the Mediterranean region based on the effect of temperature conditions on *Geobacillus stearothermophilus* growth. Food Res. Int..

[19] Kakagianni M., Gougouli M., Koutsoumanis K.P. (2016). Development and application of *Geobacillus stearothermophilus* growth model for predicting spoilage of evaporated milk. Food Microbiol..

[20] Jha S., Singh N., Anand S. (2023). Occurrence of aerobic bacterial endospores in dried dairy ingredients. Int. J. Dairy Technol..

[21] Champidou C., Ellouze M., Haddad N., Membré J.-M. (2025). Modeling *Geobacillus stearothermophilus* spores inactivation in plant-based drinks to design UHT processing. Food Res. Int..

[22] Feurhuber M., Neuschwander R., Taupitz T., Frank C., Hochenauer C., Schwarz V. (2022). Mathematically modelling the inactivation kinetics of *Geobacillus stearothermophilus* spores: Effects of sterilization environments and temperature profiles. Phys. Med..

[23] Bressuire-Isoard C., Broussolle V., Carlin F. (2018). Sporulation environment influences spore properties in *Bacillus*: Evidence and insights on underlying molecular and physiological mechanisms. FEMS Microbiol. Rev..

[24] Setlow P. (2019). Observations on Research with Spores of Bacillales and Clostridiales Species. J. Appl. Microbiol..

[25] Juneja V.K., Osoria M., Altuntas E.G., Taneja N.K., Thakur S., Kumar G.D., Setlow P. (2024). Effects of spore purity on the wet heat resistance of *Clostridium perfringens*, *Bacillus cereus* and *Bacillus subtilis* spores. Food Res. Int..

[26] Swarge B., Nafid C., Vischer N., Kramer G., Setlow P., Brul S. (2020). Investigating Synthesis of the MalS Malic Enzyme during *Bacillus subtilis* Spore Germination and Outgrowth and the Influence of Spore Maturation and Sporulation Conditions. mSphere.

[27] Ramirez-Peralta A., Zhang P., Li Y.-Q., Setlow P. (2012). Effects of Sporulation Conditions on the Germination and Germination Protein Levels of *Bacillus subtilis* Spores. Appl. Environ. Microbiol..

[28] Li L., Jin J., Hu H., Deveau I.F., Foley S.L., Chen H. (2022). Optimization of sporulation and purification methods for sporicidal efficacy assessment on *Bacillus* spores. J. Ind. Microbiol. Biotechnol..

[29] Leguérinel I., Couvert O., Mafart P. (2006). Modelling the influence of the incubation temperature upon the estimated heat resistance of heated *Bacillus* spores. Lett. Appl. Microbiol..

[30] Georget E., Kushman A., Callanan M., Ananta E., Heinz V., Mathys A. (2015). *Geobacillus stearothermophilus* ATCC 7953 spore chemical germination mechanisms in model systems. Food Control.

[31] Koransky J.R., Allen S.D., Dowell V.R. (1978). Use of ethanol for selective isolation of sporeforming microorganisms. Appl. Environ. Microbiol..

[32] Abhyankar W., Beek A.T., Dekker H., Kort R., Brul S., de Koster C.G. (2011). Gel-free proteomic identification of the *Bacillus subtilis* insoluble spore coat protein fraction. Proteomics.

[33] Begyn K., Kim T.D., Heyndrickx M., Michiels C., Aertsen A., Rajkovic A., Devlieghere F. (2020). Directed evolution by UV-C treatment of *Bacillus cereus* spores. Int. J. Food Microbiol..

[34] Zhou T., Dong Z., Setlow P., Li Y.-Q. (2013). Kinetics of Germination of Individual Spores of *Geobacillus stearothermophilus* as Measured by Raman Spectroscopy and Differential Interference Contrast Microscopy. PLoS ONE.

[35] Condón S., Arrizubieta M.J., Sala F.J. (1993). Microbial heat determinations by the multipoint system with the thermorresistometer TR-SC Improvement of this methodology. J. Microbiol. Methods.

[36] Dawson R.M.C., Elliot D.C., Elliot W.H., Jones K.M. (1974). pH, buffers and physiological media. Data for Biochemical Research.

[37] Geeraerd A.H., Herremans C.H., Van Impe J.F. (2000). Structural model requirements to describe microbial inactivation during a mild heat treatment. Int. J. Food Microbiol..

[38] Valdramidis V., Bernaerts K., Van Impe J., Geeraerd A. (2005). An Alternative Approach to Non-Log-Linear Thermal Microbial Inactivation: Modelling the Number of Log Cycles Reduction with Respect to Temperature. Food Technol. Biotechnol..

[39] Paredes-Sabja D., Setlow P., Sarker M.R. (2011). Germination of spores of Bacillales and Clostridiales species: Mechanisms and proteins involved. Trends Microbiol..

[40] Setlow P. (2013). Summer Meeting 2013—When the Sleepers Wake: The Germination of Spores of *Bacillus* Species. J. Appl. Microbiol..

[41] Burgess S.A., Flint S.H., Lindsay D., Cox M.P., Biggs P.J. (2017). Insights into the *Geobacillus stearothermophilus* species based on phylogenomic principles. BMC Microbiol..

[42] Zhao Y., Caspers M.P., Abee T., Siezen R.J., Kort R. (2012). Complete genome sequence of *Geobacillus thermoglucosidans* TNO-09.020, a thermophilic sporeformer associated with a dairy-processing environment. J. Bacteriol..

[43] Zhao Y., Kumar M., Caspers M.P.M., Nierop Groot M.N., Van Der Vossen J.M.B.M., Abee T. (2018). Short communication: Growth of dairy isolates of *Geobacillus thermoglucosidans* in skim milk depends on lactose degradation products supplied by *Anoxybacillus flavithermus* as secondary species. J. Dairy Sci..

[44] Kumar M., Flint S., Palmer J., Chanapha S., Hall C. (2021). Influence of Incubation Temperature and Total Dissolved Solids on Biofilm and Spore Formation by Dairy Isolates of *Geobacillus stearothermophilus*. Appl. Environ. Microbiol..

[45] Wang T., Flint S., Palmer J. (2019). Magnesium and calcium ions: Roles in bacterial cell attachment and biofilm structure maturation. Biofouling.

[46] Wang T., Flint S., Palmer J. (2021). Heterogeneous response of *Geobacillus stearothermophilus* biofilms to calcium. Int. Dairy J..

[47] Hyatt M.T., Levinson H.S. (1964). Effect of sugars and other carbon compounds on germination and postgerminative development of *Bacillus megaterium* spores. J. Bacteriol..

[48] Craven S.E., Blankenship L.C. (1985). Activation and injury of *Clostridium perfringens* spores by alcohols. Appl. Environ. Microbiol..

[49] Kim J., Foegeding P.M. (1990). Effects of heat-, CaCl_2_- and ethanol-treatments on activation of Bacillus spores*. J. Appl. Bacteriol..

[50] Luu S., Cruz-Mora J., Setlow B., Feeherry F.E., Doona C.J., Setlow P. (2015). The Effects of Heat Activation on *Bacillus* Spore Germination, with Nutrients or under High Pressure, with or without Various Germination Proteins. Appl. Environ. Microbiol..

[51] Berg R.W., Sandine W.E. (1970). Activation of bacterial spores. A Review. J. Food Prot..

[52] Setlow B., Loshon C.A., Genest P.C., Cowan A.E., Setlow C., Setlow P. (2002). Mechanisms of killing spores of *Bacillus subtilis* by acid, alkali and ethanol. J. Appl. Microbiol..

[53] Cronin U.P., Wilkinson M.G. (2008). Monitoring changes in germination and permeability of bacillus cereus endospores following chemical, heat and enzymatic treatments using flow cytometry. J. Rapid Methods Autom. Microbiol..

[54] Loison P., Gervais P., Perrier-Cornet J.M., Kuimova M.K. (2016). Effect of ethanol perturbation on viscosity and permeability of an inner membrane in *Bacillus subtilis* spores. Biochim. Biophys. Acta.

[55] Wang G., Chen H., Wang X., Peng L., Peng Y., Li Y.Q. (2017). Probing the germination kinetics of ethanol-treated *Bacillus thuringiensis* spores. Appl. Opt..

[56] Zhou K.X., Li N., Christie G., Wilson D.I. (2017). Assessing the Impact of Germination and Sporulation Conditions on the Adhesion of *Bacillus* Spores to Glass and Stainless Steel by Fluid Dynamic Gauging. J. Food Sci..

[57] Corcoran B.M., Stanton C., Fitzgerald G.F., Ross R.P. (2007). Growth of probiotic lactobacilli in the presence of oleic acid enhances subsequent survival in gastric juice. Microbiology.

[58] Hansen M.L., Petersen M.A., Risbo J., Hümmer M., Clausen A. (2015). Implications of modifying membrane fatty acid composition on membrane oxidation, integrity, and storage viability of freeze-dried probiotic, *Lactobacillus acidophilus* La-5. Biotechnol. Prog..

[59] Reitermayer D., Kafka T.A., Lenz C.A., Vogel R.F. (2018). Interrelation between Tween and the membrane properties and high pressure tolerance of *Lactobacillus plantarum*. BMC Microbiol..

[60] Nikiforidis C.V., Kiosseoglou V. (2007). The Role of Tween in Inhibiting Heat-Induced Destabilization of Yolk-Based Emulsions. Food Hydrocoll..

[61] Porter W.R., Staack H., Brandt K., Manning M.C. (1993). Thermal stability of low molecular weight urokinase during heat treatment. I. Effects of protein concentration, pH and ionic strength. Thromb. Res..

[62] Kanaan J., Murray J., Higgins R., Nana M., DeMarco A.M., Korza G., Setlow P. (2022). Resistance properties and the role of the inner membrane and coat of *Bacillus subtilis* spores with extreme wet heat resistance. J. Appl. Microbiol..

[63] Korza G., DePratti S., Fairchild D., Wicander J., Kanaan J., Shames H., Nichols F.C., Cowan A., Brul S., Setlow P. (2023). Expression of the 2Duf protein in wild-type *Bacillus subtilis* spores stabilizes inner membrane proteins and increases spore resistance to wet heat and hydrogen peroxide. J. Appl. Microbiol..

[64] Yu B., Kanaan J., Shames H., Wicander J., Aryal M., Li Y., Korza G., Stanley B., Kramer G., Li Y.-Q. (2023). Identification and characterization of new proteins crucial for bacterial spore resistance and germination. Front. Microbiol..

[65] Salvador M., Yruela I., Lau M.S.H., Minton N.P., Condón S., Gayán E. Identification and Role of Germinant Receptors in the Revival of *Geobacillus* and *Parageobacillus* Spores. Proceedings of the 11th European Spores Conference.

[66] Abhyankar W., Pandey R., Ter Beek A., Brul S., De Koning L.J., De Koster C.G. (2015). Reinforcement of *Bacillus subtilis* spores by cross-linking of outer coat proteins during maturation. Food Microbiol..

[67] Ghosh S., Korza G., Maciejewski M., Setlow P. (2015). Analysis of Metabolism in Dormant Spores of *Bacillus* Species by 31P Nuclear Magnetic Resonance Analysis of Low-Molecular-Weight Compounds. J. Bacteriol..

[68] Setlow P., Christie G. (2023). New Thoughts on an Old Topic: Secrets of Bacterial Spore Resistance Slowly Being Revealed. Microbiol. Mol. Biol. Rev..

[69] Sanchez-Salas J.-L., Setlow B., Zhang P., Li Y.-Q., Setlow P. (2011). Maturation of Released Spores Is Necessary for Acquisition of Full Spore Heat Resistance during *Bacillus subtilis* Sporulation. Appl. Environ. Microbiol..

[70] Camilleri E., Korza G., Green J., Yuan J., Li Y.-Q., Caimano M.J., Setlow P. (2019). Properties of Aged Spores of *Bacillus subtilis*. J. Bacteriol..

[71] Ursem R., Swarge B., Abhyankar W.R., Buncherd H., De Koning L.J., Setlow P., Brul S., Kramer G. (2021). Identification of Native Cross-Links in *Bacillus subtilis* Spore Coat Proteins. J. Proteome Res..

[72] Isticato R., Lanzilli M., Petrillo C., Donadio G., Baccigalupi L., Ricca E. (2020). *Bacillus subtilis* builds structurally and functionally different spores in response to the temperature of growth. Environ. Microbiol..

[73] Ghosh S., Setlow B., Wahome P.G., Cowan A.E., Plomp M., Malkin A.J., Setlow P. (2008). Characterization of spores of *Bacillus subtilis* that lack most coat layers. J. Bacteriol..

[74] Cowan A.E., Olivastro E.M., Koppel D.E., Loshon C.A., Setlow B., Setlow P. (2004). Lipids in the inner membrane of dormant spores of *Bacillus* species are largely immobile. Proc. Natl. Acad. Sci. USA.

[75] Garcia D., Der Voort M.V., Abee T. (2010). Comparative analysis of *Bacillus weihenstephanensis* KBAB4 spores obtained at different temperatures. Int. J. Food Microbiol..

[76] Freire V., Condón S., Gayán E. (2024). Impact of sporulation temperature on germination of *Bacillus subtilis* spores under optimal and adverse environmental conditions. Food Res. Int..

[77] Abhyankar W.R., Kamphorst K., Swarge B.N., Van Veen H., Van Der Wel N.N., Brul S., de Koster C.G., de Koning L.J. (2016). The Influence of Sporulation Conditions on the Spore Coat Protein Composition of *Bacillus subtilis* Spores. Front. Microbiol..

[78] Saggese A., Barletta G.D.G., Vittoria M., Donadio G., Isticato R., Baccigalupi L., Ricca E. (2022). CotG Mediates Spore Surface Permeability in *Bacillus subtilis*. mBio.

[79] Luo Y., Korza G., DeMarco A.M., Kuipers O.P., Li Y.Q., Setlow P. (2021). Properties of spores of *Bacillus subtilis* with or without a transposon that decreases spore germination and increases spore wet heat resistance. J. Appl. Microbiol..

[80] Planchon S., Dargaignaratz C., Levy C., Ginies C., Broussolle V., Carlin F. (2011). Spores of *Bacillus cereus* strain KBAB4 produced at 10 °C and 30 °C display variations in their properties. Food Microbiol..

[81] Trunet C., Mtimet N., Mathot A.G., Postollec F., Leguerinel I., Couvert O., Broussolle V., Carlin F., Coroller L. (2020). Suboptimal *Bacillus licheniformis* and *Bacillus weihenstephanensis* Spore Incubation Conditions Increase Heterogeneity of Spore Outgrowth Time. Appl. Environ. Microbiol..

[82] Mtimet N., Trunet C., Mathot A.-G., Venaille L., Leguérinel I., Coroller L., Couvert O. (2015). Modeling the behavior of *Geobacillus stearothermophilus* ATCC 12980 throughout its life cycle as vegetative cells or spores using growth boundaries. Food Microbiol..

[83] Baril E., Coroller L., Couvert O., Leguérinel I., Postollec F., Boulais C., Carlin F., Mafart P. (2012). Modeling heat resistance of *Bacillus weihenstephanensis* and *Bacillus licheniformis* spores as function of sporulation temperature and pH. Food Microbiol..

[84] Palop A., Mañas P., Condón S. (1999). Sporulation temperature and heat resistance of *Bacillus* spores: A review. J. Food Saf..

[85] Wells-Bennik M.H.J., Janssen P.W.M., Klaus V., Yang C., Zwietering M.H., Den Besten H.M.W. (2019). Heat resistance of spores of 18 strains of *Geobacillus stearothermophilus* and impact of culturing conditions. Int. J. Food Microbiol..

[86] Ruiz V., Alonso R., Mañas P., Condón S., Condón-Abanto S. (2022). *Geobacillus stearothermophilus* STCC4517 spore suspensions showed survival curves with shoulder phenomena independent of sporulation temperature and pH, whose duration was an exponential function of treatment temperature. Food Microbiol..

[87] Sala F.J., Ibarz P., Palop A., Raso J., Condón S. (1995). Sporulation Temperature and Heat Resistance of *Bacillus subtilis* at Different pH Values. J. Food Prot..

[88] Kumar M. (2021). A Study of the Abiotic Factors Influencing the Biofilm and Spore Formation of Dairy Isolates of *Geobacillus stearothermophilus* and Characterisation of Spores Based on Their Heat Resistance. Ph.D. Thesis.

[89] Beaman T.C., Gerhardt P. (1986). Heat resistance of bacterial spores correlated with protoplast dehydration, mineralization, and thermal adaptation. Appl. Environ. Microbiol..

[90] Melly E., Setlow P. (2001). Heat Shock Proteins Do Not Influence Wet Heat Resistance of *Bacillus subtilis* Spores. J. Bacteriol..

[91] Bressuire-Isoard C., Bornard I., Henriques A.O., Carlin F., Broussolle V. (2016). Sporulation Temperature Reveals a Requirement for CotE in the Assembly of both the Coat and Exosporium Layers of *Bacillus cereus* Spores. Appl. Environ. Microbiol..

