# Supplementary material for: Germination and Heat Resistance of Parageobacillus and Geobacillus spp. Spores
Source: Foods. 2025 Jun 11;14(12):2061. doi: 10.3390/foods14122061 (PMC12191528; doi:10.3390/foods14122061)
Supplement: Supplementary file 1 [file foods-14-02061-s001.zip › foods-3656045-supplementary.pdf]

Supplementary Material

Germination and heat resistance of *Parageobacillus* and *Geobacillus* spp. spores

Maika Salvador, Santiago Condón, Elisa Gayán

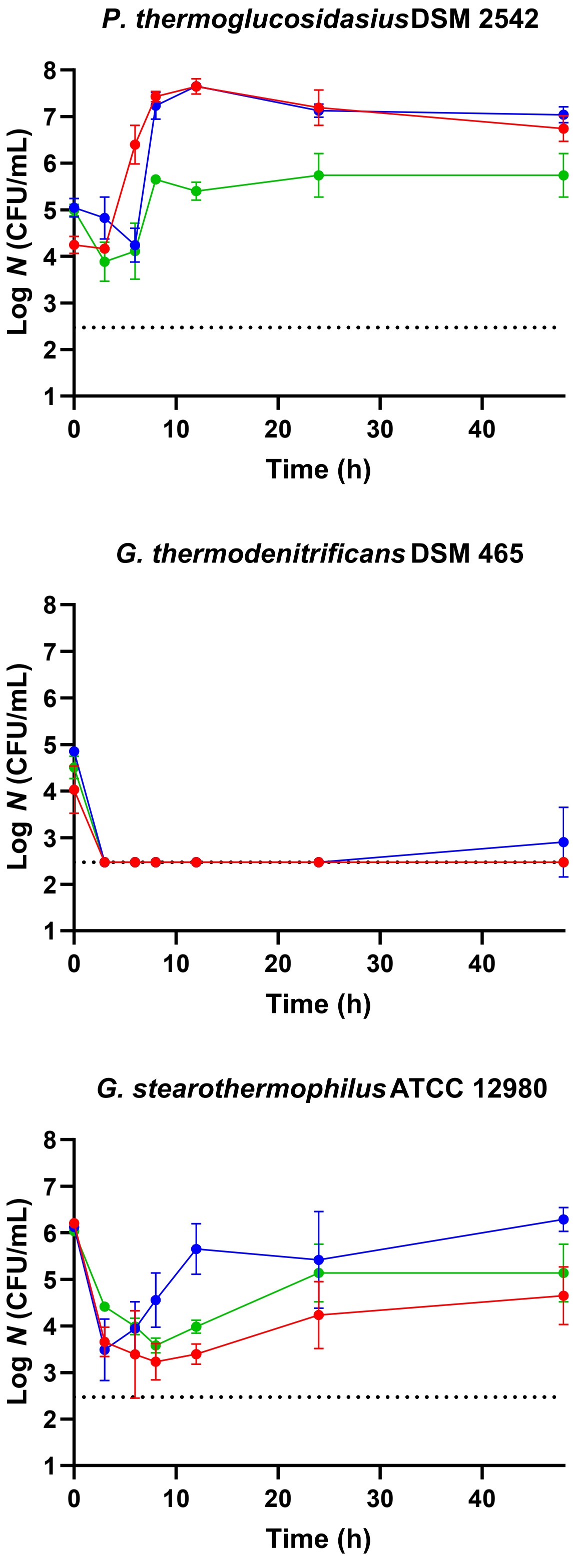

Figure S1. Growth curves of *P. thermoglucosidasius* DSM 2542, *G. thermodenitrificans* DSM 465, and *G. stearothermophilus* ATCC 12980 spores in whole milk (●), skim milk (●), and whole lactose-free milk (●) at 55 °C. Data in the figures correspond to mean values and standard deviations calculated from three biological replicates. The dotted line represents the limit of quantification (3×10^2 CFU/mL).

Table S1. Heat resistance parameters (Sl, K_{max}, and $3D_T$) of *P. thermoglucosidasius* DSM 2542, *G. thermodenitrificans* DSM 465, and *G. stearothermophilus* ATCC 12980 spores with different maturation times (1, 2, 4, or 7 d). Sporulation was performed at 55 °C and spores were purified using ethanol treatment followed by water washes (protocol 2). Data in brackets represent the standard deviations of the mean values calculated from three biological replicates.

Strain	Treatment temperature (°C)	Maturation time (d)	Sl (min)	K_{max} (min^{-1})	$3D_T$ (min)	R^2	RMSE
P. thermoglucosidasius DSM 2542	112	1	0.52^{ab} (0.47)	2.29^{a} (0.25)	3.63^{a} (0.40)	0.983	0.228
		2	0.29^{ab} (0.33)	2.20^{a} (0.23)	3.44^{a} (0.49)	0.990	0.069
		4	0.12^{b} (0.07)	2.29^{a} (0.64)	3.23^{ab} (0.75)	0.988	0.151
		7	0.60^{a} (0.26)	3.79^{b} (0.44)	2.46^{b} (0.44)	0.978	0.256
	110	1	0.62^{a} (0.97)	0.89^{a} (0.10)	9.00^{a} (0.29)	0.989	0.044
		2	0.98^{a} (0.17)	1.07^{a} (0.26)	7.55^{ab} (1.64)	0.980	0.103
		4	0.94^{a} (0.27)	1.18^{ab} (0.05)	6.93^{b} (0.82)	0.978	0.189
		7	0.73^{a} (0.50)	1.85^{b} (0.21)	4.54^{c} (0.94)	0.991	0.079
	107	1	4.81^{a} (1.48)	0.37^{a} (0.01)	23.63^{a} (1.12)	0.989	0.226
		2	1.25^{ab} (3.71)	0.33^{a} (0.06)	22.72^{ab} (0.92)	0.991	0.206
		4	1.96^{b} (0.26)	0.39^{a} (0.02)	19.77^{b} (0.91)	0.982	0.389
		7	2.38^{ab} (1.40)	0.93^{b} (0.10)	10.14^{c} (0.74)	0.980	0.289
G. thermodenitrificans DSM 465	114	1	0.42^{a} (0.39)	3.41^{a} (0.19)	2.56^{a} (0.49)	0.988	0.154
		2	0.49^{a} (0.30)	3.64^{a} (0.08)	2.45^{a} (0.29)	0.989	0.127
		4	1.25^{b} (0.16)	4.33^{b} (0.20)	2.85^{a} (0.11)	0.981	0.280
		7	0.87^{ab} (0.14)	1.92^{c} (0.19)	4.48^{b} (0.21)	0.987	0.153
	112	1	1.18^{a} (0.52)	2.03^{ab} (0.58)	4.63^{a} (0.71)	0.967	0.355
		2	1.30^{a} (0.55)	2.46^{a} (0.60)	4.16^{a} (0.24)	0.986	0.218
		4	1.96^{a} (0.61)	2.70^{a} (0.78)	4.60^{a} (0.27)	0.972	0.333
		7	1.71^{a} (1.00)	1.23^{b} (0.50)	7.58^{b} (1.97)	0.976	0.155
	110	1	2.29^{a} (0.29)	1.13^{a} (0.19)	8.46^{a} (0.83)	0.989	0.167
		2	2.34^{a} (0.09)	1.10^{a} (0.22)	8.65^{a} (1.13)	0.982	0.116
		4	2.26^{a} (0.73)	1.40^{a} (0.60)	7.23^{a} (1.87)	0.986	0.196
		7	5.62^{b} (1.34)	0.50^{a} (0.45)	13.76^{b} (1.25)	0.968	0.483
G. stearothermophilus ATCC 12980	120	1	3.41^{a} (0.98)	1.36^{a} (0.25)	8.70^{a} (0.10)	0.982	0.092
		2	2.52^{a} (0.43)	1.18^{a} (0.20)	8.47^{a} (0.71)	0.979	0.110
		4	2.15^{a} (0.86)	1.16^{a} (0.02)	8.18^{a} (1.77)	0.957	0.248
		7	2.84^{a} (0.82)	1.02^{a} (0.02)	9.63^{a} (0.77)	0.956	0.264
	118	1	6.54^{a} (0.35)	0.81^{a} (0.07)	15.14^{a} (0.53)	0.981	0.084

		2	4.03^{b} (1.39)	0.57^{b} (0.09)	16.16^{ab} (1.26)	0.974	0.126
		4	3.85^{b} (0.77)	0.63^{ab} (0.12)	14.87^{a} (0.90)	0.966	0.192
		7	5.12^{ab} (1.32)	0.53^{b} (0.02)	18.08^{b} (1.16)	0.970	0.177
	116	1	12.27^{a} (0.13)	0.43^{a} (0.11)	28.46^{a} (3.67)	0.986	0.184
		2	6.12^{b} (2.23)	0.29^{a} (0.02)	29.60^{a} (2.25)	0.980	0.250
		4	10.47^{a} (0.64)	0.35^{a} (0.03)	30.17^{a} (2.45)	0.984	0.127
		7	9.96^{ab} (1.28)	0.37^{a} (0.01)	28.60^{a} (1.73)	0.979	0.203

$^{a, b, c}$ Letters allow data to be statistically compared. In each resistant parameter, there are significant differences ($P \leq 0.05$) among spores with different maturation time within each strain and germinant when they don't share the same letter.

Table S2. Heat resistance parameters (*Sl*, K_{max}, and $3D_T$) of *P. thermoglucosidasius* DSM 2542, *G. thermodenitrificans* DSM 465, and *G. stearothermophilus* ATCC 12980 spores produced at different temperatures (50 ºC, 55 ºC, and 60 ºC). Spores were collected at day 4 and purified using ethanol treatment followed by water washes (protocol 2). Data in brackets represent the standard deviations of the mean values calculated from three biological replicates.

Strain	Treatment temperature (°C)	Sporulation temperature (°C)	*Sl* (min)	K_{max} (min^{-1})	$3D_T$ (min)	R^2	RMSE
P. thermoglucosidasius DSM 2542	112	50	0.64[a] (0.19)	4.78[a] (0.42)	2.11[a] (0.07)	0.987	0.225
		55	0.12[b] (0.07)	2.29[b] (0.64)	3.23[b] (0.75)	0.988	0.151
		60	0.00 (0.00)	1.33[c] (0.14)	5.18[c] (0.53)	0.980	0.200
	110	50	0.59[a] (0.55)	2.45[a] (0.86)	3.53[a] (0.67)	0.971	0.155
		55	0.94[a] (0.27)	1.15[b] (0.31)	6.93[b] (0.82)	0.978	0.189
		60	0.00 (0.00)	0.28[c] (0.00)	24.68[c] (0.08)	0.978	0.441
	107	50	1.25[ab] (0.59)	0.74[a] (0.16)	10.72[a] (1.58)	0.986	0.189
		55	1.96[a] (0.26)	0.39[b] (0.02)	19.77[b] (0.91)	0.982	0.389
		60	0.64[b] (0.19)	0.22[c] (0.02)	31.74[c] (3.31)	0.980	0.390
G. thermodenitrificans DSM 465	114	50	0.89[ab] (0.21)	2.97[a] (0.30)	3.24[a] (0.10)	0.979	0.263
		55	1.25[a] (0.16)	4.33[b] (0.19)	2.85[a] (0.11)	0.981	0.280
		60	0.23[b] (0.40)	1.06[c] (0.15)	7.23[b] (0.87)	0.995	0.093
	112	50	1.01[ab] (0.28)	2.52[a] (0.49)	3.81[a] (0.30)	0.981	0.230
		55	1.96[a] (0.61)	2.69[a] (0.78)	4.60[a] (0.27)	0.972	0.333
		60	0.56[b] (0.63)	0.67[b] (0.09)	10.45[b] (1.51)	0.987	0.212
	110	50	1.26[ab] (0.31)	1.10[a] (0.06)	7.59[a] (0.19)	0.975	0.135
		55	2.26[a] (0.73)	0.95[a] (0.35)	6.23[a] (1.87)	0.986	0.196
		60	0.94[b] (0.39)	0.27[b] (0.05)	26.86[b] (2.71)	0.993	0.068
G. stearothermophilus ATCC 12980	120	50	1.15[a] (0.18)	2.51[a] (0.03)	3.91[a] (0.19)	0.986	0.301
		55	2.15[a] (0.86)	1.16[b] (0.02)	8.18[b] (1.77)	0.957	0.248
		60	1.07[a] (0.17)	1.29[b] (0.16)	6.45[c] (0.48)	0.992	0.149
	118	50	2.12[a] (0.20)	1.66[a] (0.19)	7.71[a] (0.43)	0.987	0.206
		55	3.85[b] (0.77)	0.63[b] (0.12)	14.87[b] (0.90)	0.966	0.192
		60	0.54[c] (0.17)	0.53[b] (0.02)	13.30[b] (0.38)	0.993	0.151

	50	1.74[a] (1.10)	0.29[a] (0.06)	25.51[ab] (3.99)	0.979	0.106
116	55	10.47[b] (0.64)	0.35[a] (0.02)	30.17[b] (2.45)	0.984	0.127
	60	5.55[c] (1.07)	0.38[a] (0.02)	23.58[a] (0.83)	0.966	0.507

[a, b, c] Letters allow data to be statistically compared. In each resistant parameter, there are significant differences ($P \leq 0.05$) among spores prepared at different temperatures within each strain and treatment temperature when they don't share the same letter.
